# Effect of fence tray matching care on excess adhesive and bracket placement accuracy for orthodontic bonding: an in vitro study

**DOI:** 10.1186/s12903-024-04348-w

**Published:** 2024-05-12

**Authors:** Meichun Hu, Yannan Cao, Xiangbing Wu, Qian Jiang, Fangyong Zhu

**Affiliations:** 1 Wuxi Medical College, Jiangnan University, Wuxi, 214000 China; 2 Department of Stomatology, Affiliated Hospital of Jiangnan University, 1000 Hefeng Road, Wuxi, 214000 China; 3 Department of Implant Dentistry, Suzhou Stomatological Hospital, Suzhou, 215005 China; 4 Affiliated Stomatological Hospital of Guilin Medical University, Guilin, 541001 China

**Keywords:** Orthodontics, Bracket, Fence tray, Care, Excess adhesive, Transfer accuracy

## Abstract

**Objective:**

This study aimed to evaluate the effect of fence tray matching care (FTMC) in bracket bonding by measuring excess adhesive, as well as linear and angular deviations, and by comparing it with the half-wrapped tray (HWT).

**Materials and methods:**

An intraoral scanner was used to acquire data on the maxillary dental arch of a patient with periodontitis.Furthermore, 20 maxillary dental arch models were 3D printed. Using 3Shape, PlastyCAD software, and 3D printing technology, 10 FTMC (method I) and HWT (method II) were obtained. By preoperative preparation, intraoperative coordination, and postoperative measurement, the brackets were transferred from the trays to the 3D-printed maxillary dental arch models. Additionally, the bracket’s excess adhesive as well as linear and angular deviations were measured, and the differences between the two methods were analyzed.

**Results:**

Excess adhesive was observed in both methods, with FTMC showing less adhesive (*P*< 0.001), with a statistical difference. Furthermore, HWT’s vertical, tip and torque, which was significantly greater than FTMC (*P*< 0.05), with no statistical difference among other respects. The study data of incisors, canines, and premolars, showed that the premolars had more adhesive residue and were more likely to have linear and angular deviations.

**Conclusions:**

The FTMC had higher bracket bonding effect in comparison to HWT, and the adhesive residue, linear and angular deviations are smaller. The fence tray offers an intuitive view of the precise bonding of the bracket, and can remove excess adhesive to prevent white spot lesions via care, providing a different bonding method for clinical applications.

**Supplementary Information:**

The online version contains supplementary material available at10.1186/s12903-024-04348-w.

## Introduction

Bracket bonding residues often remain on the tooth surface and can easily cause white-spot lesions and bracket shedding [1–3]. Accurate positioning of the bracket is a key factor for effective orthodontic treatments. Inaccurate positioning of the bracket during the bonding process may lead to tooth deviation from the treatment direction, such as torque, rotation, and tip [4].

In 1972, Silverman et al. [5]. proposed indirect bonding (IDB) to improve the accuracy of tray placement. After the IDB of the bracket to the working model, it is transferred from the model to the tooth surface through the tray [3, 6]. Because IDB planned the placement of brackets, less clinical time was spent, and the bonding accuracy improved [7–9]. Although IDB has been advocated for many years, its routine use is influenced by additional appointments and lab work [10–12].

In 2006, Ciuffolo et al. [13]. indicated that 3D -printed trays can compensate for the reduced clinical application of IDB. The 3D printing technology allowed accurate positioning of the brackets by providing a visual assessment of the tooth root for virtual placement. Consequently, the total working time of IDB and the number of workers required were reduced through automated production [4, 14]. Moreover, 3D printing technology can design trays with different structures to meet the treatment needs of orthodontic patients [9, 10, 15–17]. Von et al. [18]. designed two different IDB transfer trays *via* 3D printing technology and validated the good accuracy and comparability of the two trays for clinical use, but did not involve adhesive.Shin et al. [19] demonstrated that 3D-printed indirect bonding tray has a slightly superior bracket placement accuracy than conventional methods, but it doesn’t improve bracket positioning accuracy. Therefore, this study aims to reduce adhesive residue and improve the accuracy of bracket bonding by designing a fence tray through 3D printing.

The fence tray’s 3D data model was obtained by scanning. Using tray design software, the bracket position of each tooth on the 3D data model was determined to design the components for locating the position of a single tooth bracket, specifically to design a component with a removable cover, single axis movement, and a cross fence that can be worn off. Furthermore, the components were copied at each tooth position on the 3D data model. The components of each tooth position were then connected by connecting pieces, and the design of the fence tray was completed by 3D printing. The fence tray uses some separate supports to provide an intuitive view of the bonding bracket, while the base design offers braced force to improve the stability and accuracy of the bonding. The separate support gives the dental assistant space to observe and operate. The dental assistant can use the scraper to remove the adhesive overflows the bracket to prevent white spot lesions and bracket shed [20, 21]. In addition, the addition of dental assistants can improve treatment effectiveness while reducing treatment time and patient discomfort [22].

Dental assistants are mostly trained nurses, who can reduce the dentist’s fatigue and soothe the patient’s mood [23]. Studies have shown that nurses assisting dentists in placing resin can improve the durability of resin use [24, 25]. When the doctor is using a fence tray, the nurse should ensure that the tray does not tilt. During tray disassembling, the nurse should pay attention to the angle and direction of the application of the doctor to reduce the shed of bracket [26].

Finally, the maxillary dental arch model of periodontitis patients was selected to compare the adhesive residue and bracket bonding accuracy of FTMC and HWT. After the condition of periodontitis is stabilized, patients often take orthodontic treatment for pathological tooth migration [27]. However, harmful bacteria in the mouth are difficult to control, and can easily recur during the orthodontic process, resulting in the interruption of orthodontic treatment [28, 29]. Thus, orthodontic treatment has higher requirements for the positioning of brackets, because the improvement of treatment effect can reduce the treatment time of patients and avoid the recurrence of periodontitis [30].Besides, excess adhesive can cause the accumulation of harmful bacteria, making it hard to effectively remove plaque through brushing teeth [31].Designing the fence tray, we hope to improve the efficacy and application of orthodontics in various populations.

This paper compares the use of FTMC and HWT by measuring excess adhesive, linear and angular deviations. The null hypothesis was that there is no difference in excess adhesive, linear and angular deviations between FTMC and HWT.

## Materials and methods

This in vitro study was approved by the Medical Ethics Committee of the Affiliated Hospital of Jiangnan University in October 2022 (approval number: LS2022106). Informed consent of the patient has been obtained. Firstly, select a patient with periodontitis and acquire the digital impression of the maxillary dental arch. The inclusion criteria for this study were: (1)complete maxillary dentition, (2) normal tooth structure, (3) no orthodontic treatment history, (4) stable period of periodontitis. Exclusion criteria: (1) caries, (2) implant implantation, (3) poor oral hygiene, (4) dental deformities and quantitative defects, (5) severe tooth displacement hinders the placement of the tray.The sample size was estimated by G*Power (version 3.1.9.4.) according to previous studys [4, 32, 33],with an analysis [90% power (Cohen’s d = 0.5); 5% significance level; Mann-Whitney U tests (two-tailed)]. Therefore, each group should use at least 10 maxillary dental arches to detect the difference between the two methods.

### Preoperative preparation

**Fig. 1:**
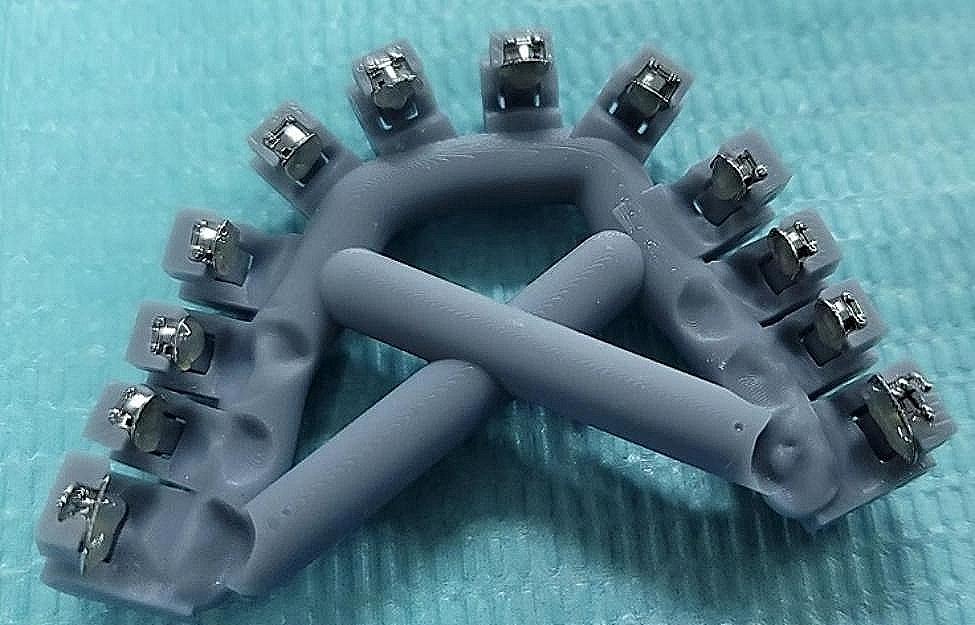
Fence tray

**Fig. 2:**
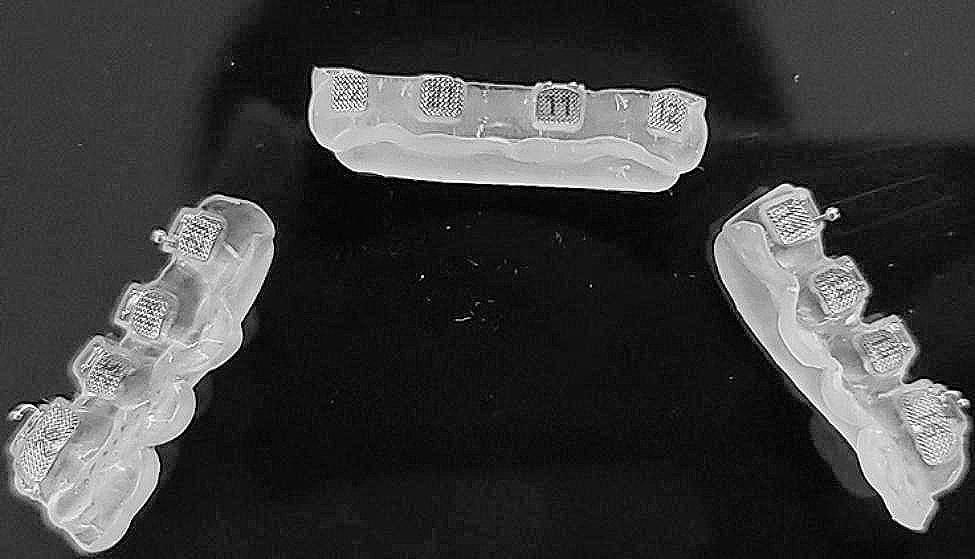
Half-wrapped tray

OrthoAnalyze™ (3Shape; Copenhagen, Denmark) software makes 20 maxillary dental arch models. Afterward, the bracket position was set virtually based on the model. Subsequently, 10 spare pairs of fence trays (Fig. 1) and HWT (Fig. 2) were designed and printed using 3shape and PlastyCAD software.

Instrument preparation: The maxillary dental arch model, bracket [Mini 0.022MBT (Protect Orthodontics Mini MBT 022 with hooks 5 to 5)], transfer tray, conventional treatment plate, instrument, light curing lamp, 3 M light curing adhesive (3 M Unitek Transbond™ MIP), and separating agent (3 M Unitek Transbond™ MIP) were prepared for subsequent treatment.

### Intraoperative cooperation

**Fig. 3:**
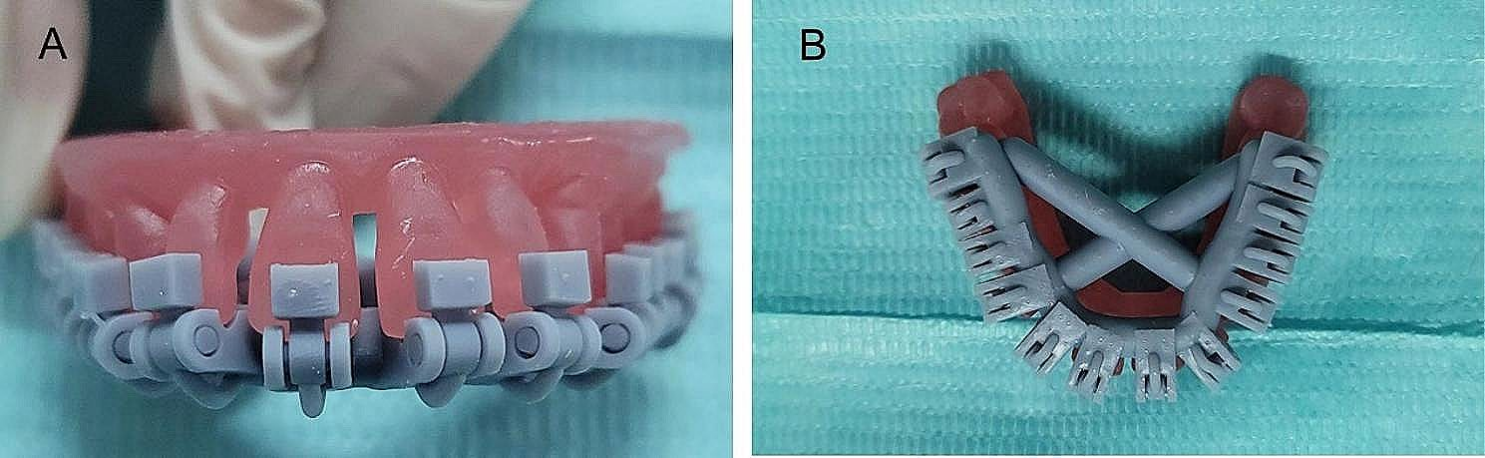
Indirect bonding effect of fence tray: front view **(A)**, top view **(B)**

**Fig. 4:**
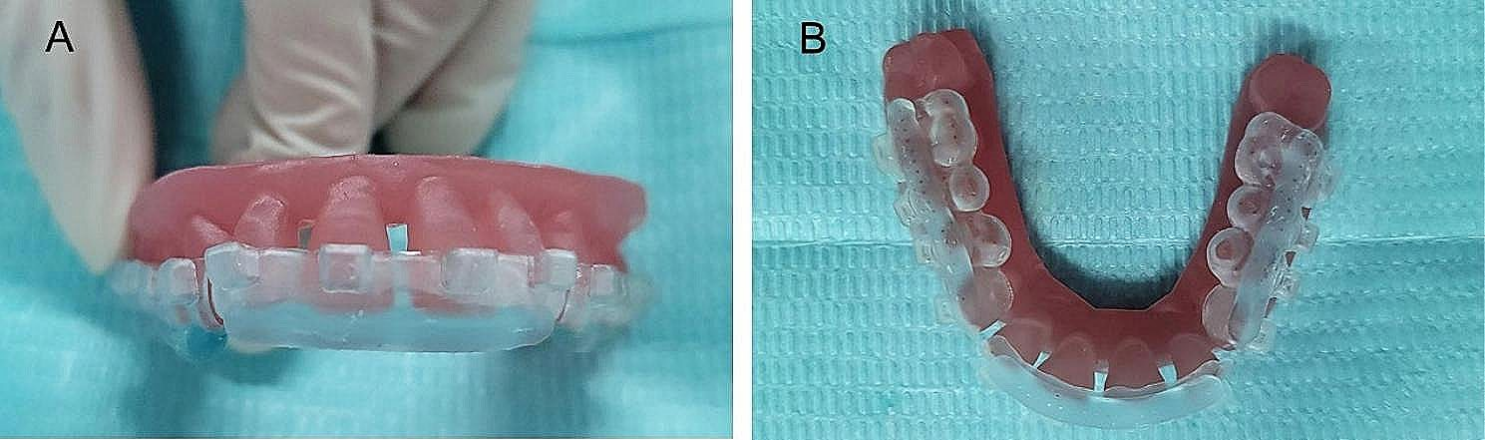
Indirect bonding effect of half-wrapped tray: front view **(A)**, top view **(B)**

First, the tray pre-coated with the separating agent is dried, and then the bracket pre-coated with the adhesive is placed. During the bonding process, the nurse ensured that the tray did not tilt. The bracket was bonded accurately and light-cured for 10 s. Furthermore, the excess adhesive around the fence tray were removed using a scraper. The doctor performed the HWT bonding alone. The IDB’s effects on the fence tray and the HWT are shown in Figs. 3 and 4, respectively. Before removing the tray, the nurse soaked the tray in water to remove the separating agent.

### Postoperative measurement

The digital model of the maxillary dentition, including the bracket was transferred to the virtual reality *via* a Reco CS3600 intraoral scanner with an accuracy of 20 μm and Standard Tessellation Language (STL) output format. Then the STL files were imported into Medit T500 (Medit, Seoul, Korea) for automatic surface registration using a virtual model based on an iterative closest point matching algorithm. An operator with 5 years of experience with Medit is responsible for all measurements.

**Fig. 5:**
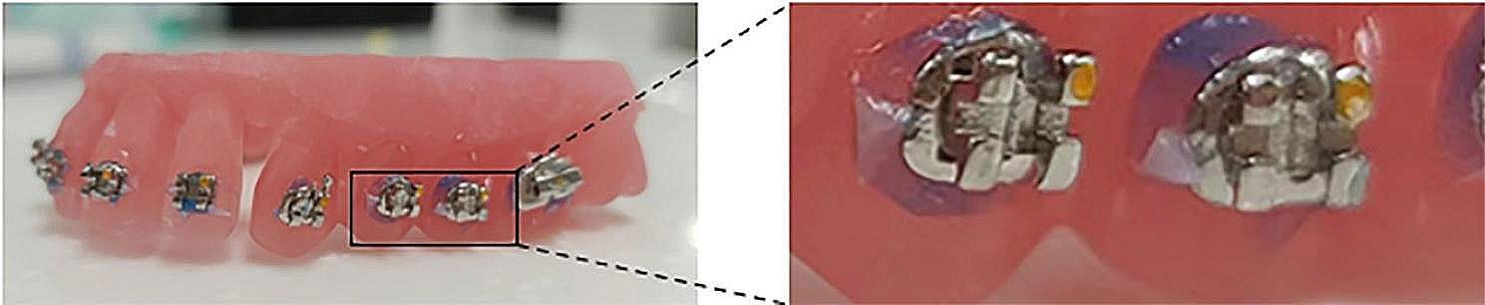
Residual adhesive around the bracket

**Fig. 6:**
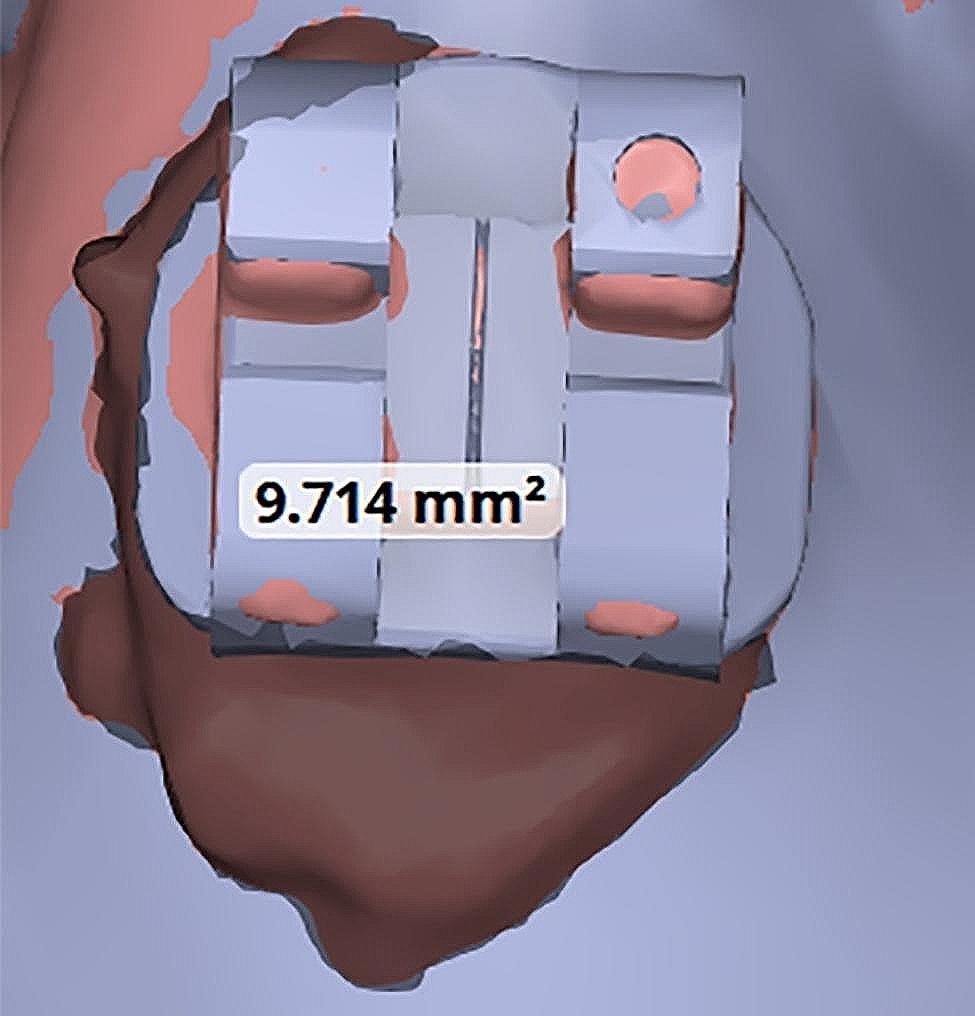
Measure excess adhesive around the bracket

Measurement of the excess adhesive: The adhesive residue was clearly visible around the brackets of the actual models (Fig. 5). The excess adhesive around the brackets of the virtual model was measured using the Medit T500’s area measurement function (Fig. 6).

**Fig. 7:**
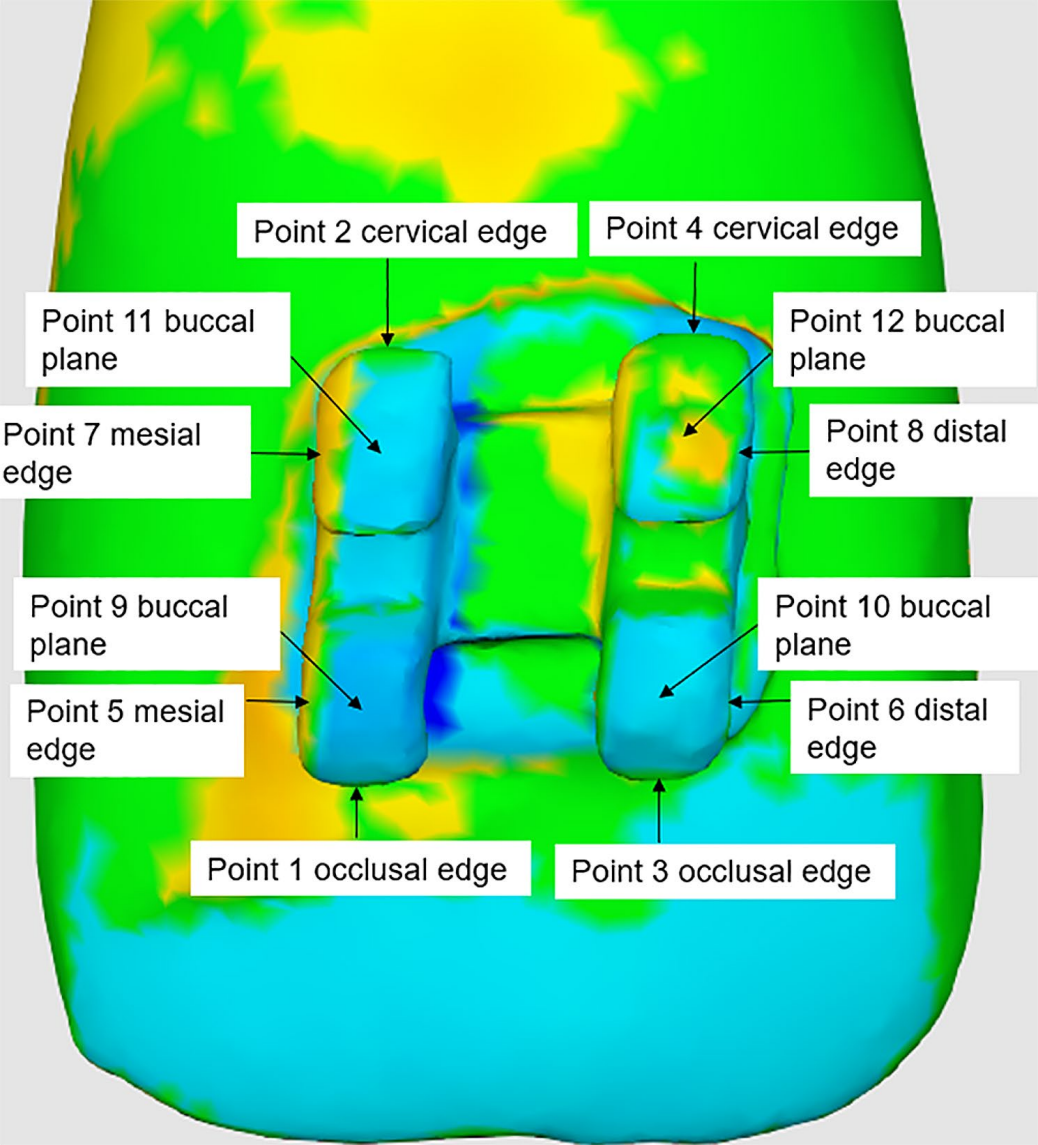
The 12 measuring points

Measurement of linear and angular deviations: To calculate the occlusal-cervical (vertical), mesio-distal (horizontal), and buck-lingual (transversal) directions, as well as in tip, rotation, and torque, 12 points were marked on the virtual model (Fig. 7).

As Fig. 7 indicates, the Medit T500 was used to measure the values of each four points and their average, which represents the linear deviations generated in the vertical, horizontal, and transversal directions. For angular deviations, two corresponding points of the actual and virtual models were measured *via* software. The rotation was assessed by calculating the mean of the angle formed by points 1 and 3 as well as points 2 and 4. The torque was measured by calculating the mean of the angle formed by points 5 and 7 as well as points 6 and 8, the tip was elucidated by assessing the mean of the angle formed by points 9 and 12 as well as points 11 and 10.

**Fig. 8:**
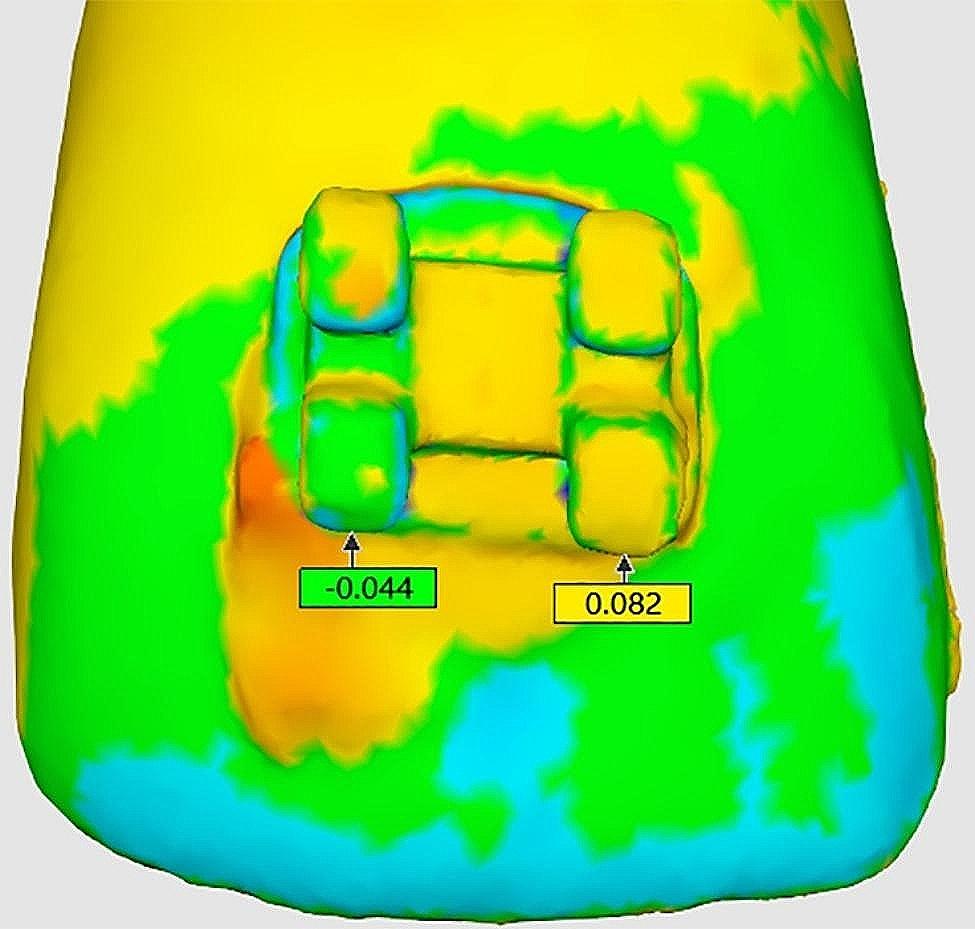
Measurement of the linear deviation (vertical direction) of the bracket

**Fig. 9:**
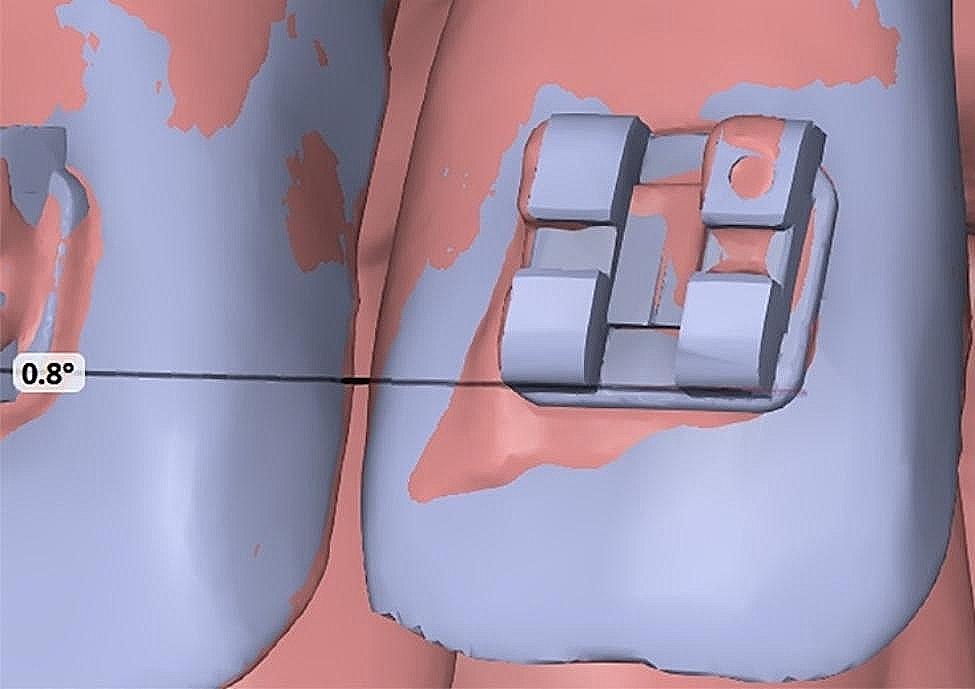
Measurement of the angular deviation (rotation) of the bracket

For linear deviation, a positive value indicated that the actual models were exposed more, while a negative value indicated greater exposure to the virtual model. For example, the values for points 1 and 3 were 0.038 mm, indicating that the actual models of the bracket had shifted in an occlusal direction (Fig. 8). Furthermore, for the angular deviation, the angle formed by the lines at points 1 and 3 for the two models indicated that the unilateral rotation error of the bracket was 0.8° (Fig. 9).

### Statistical analysis

The data did not have a normal distribution. The median [interquartile range (IQR)] was used to describe the transfer deviation between the two groups. The overall significant difference in transfer deviations between the three tooth groups (incisors, canines, premolars) was assessed by the non-parametric Kruskal-Wallis H multipair comparison. Furthermore, the differences between the two transfer methods were determined using the Mann-Whitney test. The *p*-value < 0.05 was considered statistically significant. All statistical analyses were performed using SPSS v26.0.

## Results

A total of 6 and 8 brackets in methods 1 and 2 fell off during the transfer process, respectively, and therefore, were not included in the evaluation. This study determined 186 brackets (94 in method 1 and 92 in method 2). Tables 1 and 2 show that both methods have adhesive residue, prominent linear deviation in the vertical direction, and the worst bonding accuracy in the torque direction in the angular deviation. Furthermore, here, the linear and angular measurements of 0.25 mm and 1°, respectively, were set as clinically acceptable limits. Although method 2 indicated a greater residual adhesive as well as linear and angular deviations than method 1, the deviation of method 2 was still clinically acceptable.

The comparison of the bonding effect of the three tooth groups revealed that all the tooth groups of method 1 and method 2 had adhesive residue, with the premolars having the most adhesive residue. Moreover, the error of premolars in the vertical direction was the largest in method 1. There were no significant differences in other groups (Table 1). Although method 2 vertical direction exhibited differences among the groups, the Kruskal-Wallis H had no differences. The transfer deviation of the incisor in the transversal direction was small, but the rotation was large. Additionally, premolars also exhibited substantial deviations in torque (Table 2).

The comparison of the two methods revealed that the excess adhesive in method 2 [9.673 (8.185–11.237)] was higher than those in method 1 (*P*< 0.001), and the difference was statistically significant. For linear deviation, the vertical deviation of method 2 [0.191 (0.136–0.210)] was statistically significantly greater than method 1 (*P*< 0.001). Furthermore, in angular deviation, the values of method 2 tip [0.788 (0.717–0.825) (*p*< 0.05)] and torque [0.908 (0.855–0.983) (*p*< 0.001)] were more than method 1, and the difference was statistically significant. The transfer errors of horizontal, transversal, and rotational methods were similar and were not statistically significant. Altogether, the data indicated that method 1 had less adhesive residue and higher bonding accuracy than method 2 (Table 3).

**Table 1:** Method 1 (FTMC) comparison of excess adhesive, linear and angular deviations in incisors, canines and premolars

Variables	Median (IQR)				Overall difference between tooth groups (*p*-values)	Pairwise comparisons (*p*-values)
	Incisors (I) *N* = 38	Canines (C) *N* = 19	Premolars (P) *N* = 37	All Groups *N* = 94		
Excess adhesive (mm^2^)	5.438(4.210-6.849)	5.736(4.161-7.843)	7.031(5.519-9.311)	6.014(4.300-8.207)	< 0.05*	I/P:<0.05*
Vertical (mm)	0.130(0.112-0.154)	0.148(0.108-0.154)	0.154(0.137-0.180)	0.146(0.116-0.156)	< 0.05*	I/*P*< 0.05*
Horizontal (mm)	0.101(0.087-0.136)	0.098(0.089-0.121)	0.092(0.087-0.097)	0.096(0.087-0.115)	0.137	-
Transversal (mm)	0.086(0.082-0.094)	0.093(0.082-0.099)	0.087(0.081-0.094)	0.087(0.082-0.094)	0.742	-
Tip (°)	0.748(0.708-0.791)	0.732(0.661-0.788)	0.718(0.665-0.760)	0.742(0.686-0.777)	0.424	-
Rotation (°)	0.813(0.788-0.861)	0.801(0.756-0.842)	0.810(0.795-0.853)	0.807(0.787-0.857)	0.529	-
Torque (°)	0.821(0.795-0.881)	0.838(0.801-0.864)	0.848(0.785-0.886)	0.836(0.796-0.878)	0.973	-

IQR: interquartile range, I: Incisors, C: Canines, P: Premolars, *Significant difference (*P*< 0.05)

**Table 2:** Method 2 (HWT) comparison of excess adhesive, linear and angular deviations in incisors, canines and premolars

Variables	Median (IQR)				Overall difference between tooth groups (*p*-values)	Pairwise comparisons (*p*-values)
	Incisors (I) *N* = 34	Canines (C) *N* = 20	Premolars (P) *N* = 38	All Groups *N* = 92		
Excess adhesive (mm^2^)	8.676(7.636-10.255)	9.214(8.045-11.535)	10.634(9.620-12.137)	9.673(8.185-11.237)	< 0.05*	I/P:<0.05*
Vertical (mm)	0.202(0.146-0.220)	0.206(0.121-0.232)	0.179(0.109-0.201)	0.190(0.136-0.210)	< 0.05*	-
Horizontal (mm)	0.097(0.076-0.120)	0.090(0.069-0.153)	0.091(0.072-0.103)	0.092(0.072-0.118)	0.621	-
Transversal (mm)	0.076(0.054-0.092)	0.099(0.088-0.130)	0.101(0.090-0.114)	0.095(0.070-0.108)	< 0.05*	I/P:<0.05* I/C:<0.05*
Tip (°)	0.815(0.747-0.842)	0.788(0.734-0.820)	0.754(0.610-0.814)	0.788(0.717-0.825)	0.057	-
Rotation (°)	0.840(0.795-0.859)	0.794(0.615-0.840)	0.796(0.701-0.819)	0.802(0.731-0.847)	< 0.05*	P/I:<0.05*
Torque (°)	0.856(0.794-0.898)	0.901(0.879-0.969)	0.945(0.912-1.202)	0.908(0.855-0.983)	< 0.001***	I/P: <0.001***

IQR: interquartile range, I: Incisors, C: Canines, P: Premolars, *Significant difference (*P*< 0.05), ***Significant difference(*P*< 0.001)

**Table 3:** Compare the excess adhesive, linear and angular deviations generated by Method 1 and Method 2

Variables	Median (IQR)		*p*-values
	Method 1 (FTMC) *N* = 94	Method 2 (HWT) *N* = 92	
Excess adhesive (mm^2^)	6.014(4.300-8.207)	9.673(8.185-11.237)	< 0.001***
Vertical (mm)	0.146(0.116-0.156)	0.191(0.136-0.210)	< 0.001***
Horizontal (mm)	0.096(0.087-0.115)	0.092(0.072-0.118)	0.058
Transversal (mm)	0.087(0.082-0.094)	0.095(0.070-0.108)	0.146
Tip (°)	0.742(0.686-0.777)	0.788(0.717-0.825)	< 0.05*
Rotation (°)	0.807(0.787-0.857)	0.802(0.731-0.847)	0.067
Torque (°)	0.836(0.796-0.878)	0.908(0.855-0.983)	< 0.001***

IQR: interquartile range, FTMC: fence tray matching care, HWT: half-wrapped tray, *Significant difference (*P*< 0.05), ***Significant difference(*P*< 0.001)

## Discussion

This study aimed at evaluating the effects of FTMC and HWT on the bonding effect of brackets through excess adhesive, linear and angular deviations.The results indicated that FTMC is significantly different from HWT in terms of excess adhesive, vertical, tip, and torque, with FTMC shows less adhesive and smaller transfer deviations.The null hypothesis presented, that no difference in excess adhesive, linear and angular deviations between FTMC and HWT, was rejected.

In this study, because of the uneven tooth surface, insufficient curing time, and incomplete scanner capture after brackets transfer to the tooth surface, some brackets did not meet the measurement conditions as they fell off in both methods. Therefore, 94 and 92 brackets were measured with methods 1 and 2, respectively. To reduce the error of manual measurement, an operator with 5 years of experience with Medit was selected to measure excess adhesive, linear and angular deviations, and calculated the mean.The results of this study can be used as a reference for other systems as the mini bracket used is similar to other bracket systems in design and size.

Currently, the acceptance criteria for deviations generated by brackets vary. The American Orthodontic Board (ABO) uses linear deviations ≤ 0.5 mm and angular deviations ≤ 2 ° as standards [34]. Schmid et al. [16]. suggested that two adjacent brackets deviate in opposite directions and set 0.25 mm and 1° as the acceptable limits of incisor, canine, and premolar. Armstrong et al. [35]. indicated that the linear deviation of incisors within 0.25 mm and other types of teeth within 0.5 mm was clinically significant. Considering the different linear and angular deviation setting standards, ≤ 0.25 mm and ≤ 1°, respectively, were set as the acceptable limits in this study.

Compared with the excess adhesive, both types of trays were most obvious in premolars, which might be because it is difficult to clean the premolars as they were located in the inner part of the mouth, and a large adhesive area was used. There were significant differences in excess adhesive between the two methods, and the residual adhesive in the FTMC was small. This is potential because, during FTMC, the nurse uses a scraper to remove the excess adhesive around the bracket. A comparison of this study with the research of Mohlhenrich [3] et al. revealed that the transfer tray’s structure impacts the adhesive residual. Thus, the fence tray in this study not only increased the bracket’s bonding accuracy but also took better care of the tray structure, reducing the adhesive residue.

In linear and angular measurements, the vertical deviation of the FTMC and the torque of the HWT were the most prominent in the premolars. The linear and angular deviations of the FTMC were not significantly different in other tooth types. The largest deviation in HWT rotation was observed in the incisors. The transversal direction was less deviated in the incisors and most obvious in canines and premolars. Errors in premolars may be caused by the teeth being located at the back of the mouth, which has limited operating space [8, 18, 36]. Whereas the errors in canines are usually caused by their convex surface and limited bonding range. Although incisors show high bonding accuracy, they are prone to deviation in rotation. These data are consistent with Scisciola et al. [37] and Gundoğ et al. [38]. Furthermore, these deviations may be associated with tooth morphology or unavoidable factors during the operation.

The comparison of linear measurements indicated that vertical direction was the most evident deviation in the two methods, especially the HWT.The linear measurements of both methods are ≤ 0.25 mm, consistent with the data of Castilla et al. [39] and Palone et al. [40]. Compared with angular measurements, there were deviations in torque and tip between the two methods, with torque deviation being significantly larger. The FTMC showed a small deviation, with both angle measurements ≤ 1°. Moreover, there were no statistically significant differences in the horizontal, transversal, and rotation of the two methods.The different transfer deviations of the two kinds of trays can be due to the HWT’s segmented design, as it has a large positioning path error and does not require care. In contrast, the fence tray adopts an independent support and base design. The care ensures accurate bracket bonding positioning during the transfer process and avoids deviation during tray bonding.

The fence tray, a new method of bonding brackets, offers new ideas for transfer tray design. FTMC enhances the preciseness of bracket bonding and diminishes adhesive residue to prevent white spot lesions [41]. Furthermore, the fence tray’s design makes medical cooperation easier, alleviates muscle soreness caused by doctors operating alone, and shortens patient treatment time [42]. The selection of the maxillary dental arch model of periodontitis patients for experimentation is to demonstrate that FTMC can meet the requirements of periodontitis patients for the accuracy of bracket bonding, and expand the application of fence trays in different populations. Maintaining oral hygiene of periodontitis patients and reducing pathogen aggregation can be achieved by reducing adhesive residue after FTMC bonding [43]. Patients with periodontitis can receive effective orthodontic treatment through the use of a fence tray in a clinic.

The limitation of this study is that it was an in vitro study and does not consider the influence of the in vivo environment on tray bonding, such as the limited range of operation in the mouth leading to more difficult transfer, salivary interference, and patient health. Therefore, follow-up in vivo experiments should be conducted to better evaluate the effectiveness of the FTMC. The linear and angular deviation assessment, bracket bonding, and the measurement of excess adhesive were carried out manually, which caused inevitable errors in the study.

## Conclusion

FTMC and HWT both have the capability to place brackets accurately, but FTMC has less adhesive, smaller vertical, tip, and torque deviations, and no significant differences in other aspects. According to the research results, the fence tray can decrease adhesive residue and enhance the precision of bracket placement, making it a superior method for IDB.

### Electronic supplementary material

Below is the link to the electronic supplementary material.

Supplementary Material 1

Supplementary Material 2

## Data Availability

The datasets generated and analyzed during the current study are available from the corresponding author on reasonable request.

## Abbreviations

FTMC: Fence tray matching care
HWT: Half-wrapped tray
IDB: Indirect bonding
STL: Standard Tessellation Language
IQR: Interquartile range
I: Incisors
C: Canines
P: Premolars
ABO: The American Orthodontic Board
